# Supplementary material for: Feature selection strategies for drug sensitivity prediction
Source: Sci Rep. 2020 Jun 10;10:9377. doi: 10.1038/s41598-020-65927-9 (PMC7287073; doi:10.1038/s41598-020-65927-9)
Supplement: Supplementary file 1 — Supplementary Information. [file 41598_2020_65927_MOESM1_ESM.pdf]

Feature selection strategies for drug sensitivity prediction
Supplementary Information

Krzysztof Koras[1], Dilafruz Juraeva[2], Julian Kreis[2], Johanna Mazur[2], Eike Staub[2], and Ewa Szczurek[1,*]

[1]Faculty of Mathematics, Informatics and Mechanics, University of Warsaw
[2]Merck KGaA, Translational Medicine, Department of Bioinformatics
[*]szczurek@mimuw.edu.pl

1 Supplementary Methods

1.1 Elastic net regression

Elastic net regression belongs to the family of regularized linear regression models where the target value is expected to be a noisy linear combination of input features. The model introduces the regularization through adding a combination of ℓ_1 and ℓ_2 norms of it's coefficients to the loss function. Therefore, elastic net's optimization problem can be represented as:

$$\min_{w} \frac{1}{n} \|Xw - y\|_2^2 + \alpha\rho\|w\|_1 + \alpha(1-\rho)\|w\|_2^2$$

where n is the number of samples, X and w represent the input data and coefficients vector, respectively. The amount and type of regularization are controlled by hyperparameters α and ρ, corresponding to *alpha* and *l1_ratio* arguments in scikit-learn implementation[1]. These two parameters where tuned during cross-validation.

1.2 Random forest regression

Random forest is an ensemble method, which works by combining the outputs of several decision trees in order to make final predictions. In contrast to linear regression, decision trees is a non-parametric method which learns simple decision rules inferred from the data features. It's goal is to partition the input feature space such that samples with similar labels are grouped together. At each node m, corresponding data Q is split into two subsets:

$$\begin{aligned} Q_{left}(\theta) &= (x,y)|x_j < t_m \\ Q_{right}(\theta) &= Q \setminus Q_{left} \end{aligned}$$

where θ is a candidate split consisting of a feature j and corresponding threshold t_m and (x,y) represents training samples. Decision trees select parameters j and t_m which minimize the impurity of resulting subsets. The choice of a specific impurity function depends on application. In our analysis, we used mean squared error, which is common for regression tasks.

Decision trees have many advantages, but are also prone to create over-complex graphs which tend to overfit the data. In random forest, each tree is built from the bootstrap sample from the training set. Furthermore, the best split at each node is picked from a random subset of features. Such randomness, combined with averaging the predictions of single trees, helps to decrease the variance of the overall model. The hyperparameters we tuned when using random forests included (following scikit-learn notation): *n_estimators* – number of trees in the ensemble, *max_features* – maximum number of features considered when splitting the data, *max_depth* – maximum depth of the trees, *min_samples_split* – minimum number of samples required to perform the split and *min_samples_leaf* – minimum number of samples allowed in a leaf node.

1.3 Stability selection with lasso regression.

Stability selection[2] works by generating N bootstrap samples of available data and using an underlying feature selection algorithm (in this case lasso regression) to determine which features are relevant for a given sample. For every generated sample, it fits the selection algorithm with a specified value of regularization parameter λ, which produces a selection set $\hat{S}_i^\lambda$ indicating which features to choose. Given selection sets from each sample, the empirical probability of choosing a particular feature k can be computed as:

$$\hat{\Pi}_k^\lambda = \Pr[k \in \hat{S}^\lambda] = \frac{1}{N}\sum_{i=1}^{N} \mathbb{I}_{\{k \in \hat{S}_i^\lambda\}}$$

i.e. counting the number of times k occurred as an important component for in the samples. This process is then repeated for several values of λ. The final stable set of relevant features can be then constructed as follows:

$$\hat{S}^{\text{stable}} = \{k : \max_{\lambda \in \Lambda} \hat{\Pi}_k^\lambda \geq \pi_{\text{thr}}\}$$

where Λ is a set of all λ values and π_{thr} is a predefined probability threshold. In our work, we used scikit-learn compatible implementation of stability selection[3] combined with lasso regression, fitting for $N = 100$ samples with five different values of λ: $10^{-5}, 10^{-4}, 5 \cdot 10^{-4}, 10^{-3}$ and 10^{-2}.

When applying automated stability selection, we first fitted the model using five different values of λ and 100 bootstrap samples, which resulted in stability scores corresponding to every feature. We then iterated over predefined range (0, 1) of stability thresholds π_{thr} with 0.025 increment, performing the whole modeling process with a corresponding number of features at each iteration using elastic net regression. This procedure was repeated for five random data splits. In order to establish the single best stability threshold for every compound, we averaged the results over data splits. The performance metrics used to evaluate the model were then the averages of metrics achieved with the chosen best threshold for every data split.

1.4 Feature importance derived from random forest

In a single decision tree, the depth of a feature used as a decision node represents the relative importance of that feature when predicting the target variable. Features present at the top of a tree contribute to the final prediction result for a bigger fraction of samples. The importance of a particular feature is also associated with the decrease of impurity when splitting the data using that feature (i.e. the bigger the importance, the bigger decrease in impurity measure). Therefore, the corresponding impurity decrease can be used to estimate the feature importance in a single tree[4]. In random forests, this predictive ability of a given feature can be averaged over several trees to define a new metric, *Mean Decrease Impurity* (MDI)[5], which provides the feature importance estimate with reduced variance.

When using random forest for feature selection, after data extraction and hyperparameter tuning steps, we first trained the algorithm on the whole training set and extracted a vector with values representing the importance of all features. We then ranged over a grid of values k, each time performing the whole modeling process using random forests regression with k most important features and recording the corresponding results. Similarly, as in the stability selection setting, one best value of k was chosen by averaging the results over five data splits, and the corresponding performance metrics for best found k were averaged in order to evaluate the model.

References

1. Pedregosa, F. *et al.* Scikit-learn: Machine learning in Python. *J. Mach. Learn. Res.* **12**, 2825–2830 (2011).
2. Meinshausen, N. & Bühlmann, P. Stability selection. *J. Royal Stat. Soc. Ser. B (Statistical Methodol.* **72**, 417–473 (2010).
3. stability_selection module (2019). https://thuijskens.github.io/stability-selection/docs/index.html.
4. Louppe, G. *Understanding Random Forests: From Theory to Practice*. Ph.D. thesis, University of Liège, Faculty of Applied Sciences (2014).
5. Louppe, G., Wehenkel, L., Sutera, A. & Geurts, P. Understanding variable importances in forests of randomized trees. In Burges, C. J. C., Bottou, L., Welling, M., Ghahramani, Z. & Weinberger, K. Q. (eds.) *Advances in Neural Information Processing Systems 26*, 431–439 (Curran Associates, Inc., 2013).

2 Supplementary Figures

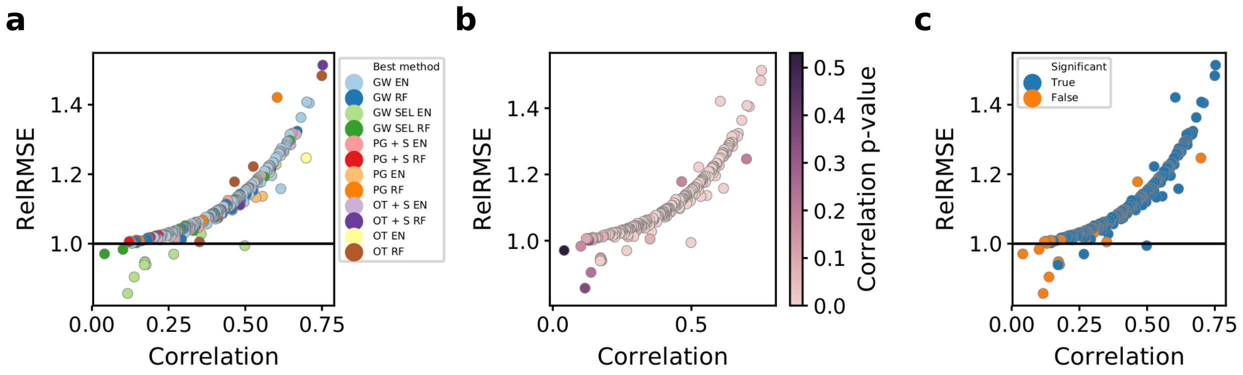

Figure S1. P-values of achieved correlations with the test set, calculated based on Student's t-distribution. (**a**) RelRMSE vs. correlation obtained by the best model for a given drug (copy of Fig. 3c from the main manuscript for reference). (**b**) Same plot as in panel **a**, colored by the corresponding correlation p-value. (**c**) Same plot as in panel **a**, with corresponding correlation p-values classified into significant and non-significant categories at 0.05 confidence level. See Fig. 1 in the main manuscript for model abbreviations.

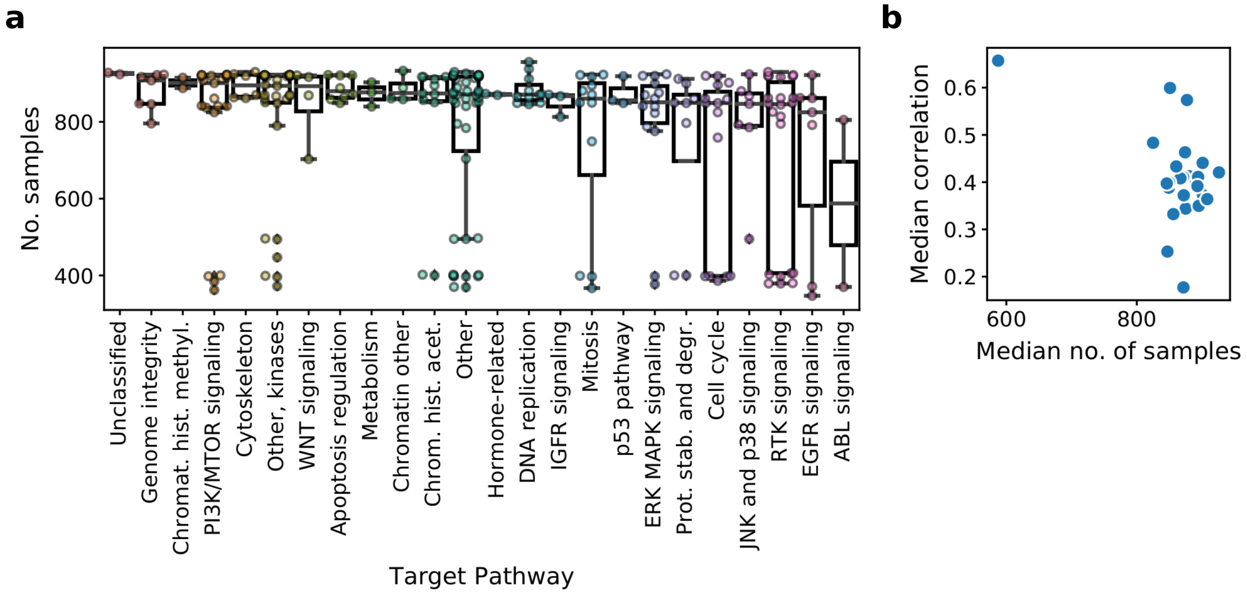

Figure S2. Data availability and modeling perfromance grouped by target pathways of the drugs. (**a**) Number of available samples per drugs belonging to a specific target pathway. Target pathways are sorted by median of per-drug samples. The median values are similar across target pathways (excluding ABL signaling pathway), however, with some pathways exhibiting significant spread. (**b**) Median modeling performance versus median number of per-drug available samples across drugs belonging to given pathways (each point represents a specific target pathway).

3 Supplementary Tables

Supplementary Table S1

Gene expression signatures along with corresponding number of genes and reference. For signatures with missing references, list of genes is provided below the table.

Signature Name	Genes	PMID
Kannengiesser.BRAF.signature	25	19383316
IFN_signature	32	
KinetochoreNet	33	
B_cell_signature_IRIS	90	
T_cell_signature_IRIS	14	
DNAsynthesisFuncNet	64	
Chi.Hypoxia.BrownVanDeVijver	18	16417408
Breast_ERBB2subtype.Calza2006	8	16846532
Breast_BASALsubtype.Calza2006	17	16846532
Breast_LuminalBsubtype.Calza2006	9	16846532
ER_pos_BreastCa.Abba2005	9	15762987
TGFbeta.EarlyUp.Verrecchia2001	50	11279127
TNFa_NFkB_pw_response.Tian2005	20	15722553
B_cell_signature.Newell2010.markers	18	20501946
medulloblastoma.IFN.up.Staub2012	10	22937184
medulloblastoma.PRF.up.Staub2012	21	22937185
BreastCaCellCycleSig.Dai2005	36	15899795
BreastStromaMetagene.Farmer2005	50	15897907
AKT.up.Creighton2007	57	17213801
EMT.BreastCa.Lien2007	33	17603561
ClassicalPancreasCa.Collison2011	22	21460849
Phillips2006.ProNeural	15	16530701
Phillips2006.Prolif	5	16530702
Phillips2006.Mesenchymal	15	16530703
Lottaz2010.TypeII.CSC	21	20145155
Lottaz2010.TypeI.CSC	8	20145156
Freije2004.GBM.prognGroup.HC2A	10	15374963
Freije2004.GBM.prognGroup.HC2B	15	15374964
CDK8_genomic_neighbors	6	
Liang2005.GBM-Hypoxia	21	15827124
Liang2005.GBM-ECM	19	15827125
Liang2005.GBM-Prolif	22	15827126
Farmer2005.BreastCa.Clu1.IFN_Tcell_Bcell	44	15897900
Farmer2005.BreastCa.Clu2.prolif_8qamplicon	33	15897901
Farmer2005.BreastCa.Clu3.apocrine_basal_hypoxia	15	15897902
Farmer2005.BreastCa.Clu5.17q21_32amplicon	17	15897903
Farmer2005.BreastCa.Clu6.luminal	16	15897904
Farmer2005.BreastCa.Clu7.apocrine_luminal	20	15897905
Farmer2005.BreastCa.Clu8.ERBB2amplicon	7	15897906

Farmer2005.BreastCa.Clu4.stroma	19	15897907
EMTup.QiagenPCRarray	36	
Kuner2009.LungSCCvsAD	10	18486272
Cytosolic.Ribosomal.Proteins	68	
Sadanandam2013_InflammatoryCRC	16	23584089
Sadanandam2013_StemLikeCRC	164	23584093
Wilkerson2010_LungSqCC_PrimitiveSubtype	30	20643781
Wilkerson2010_LungSqCC_ClassicalSubtype	18	20643781
Wilkerson2010_LungSqCC_SecretorySubtype	24	20643781
Wilkerson2010_LungSqCC_BasalSubtype	17	20643781
SomaLogic_PlateletActivationInPlasma	15	
ECM_qPCR_panel_Qiagen	83	
Feng2006_IFN_5gSig	5	16947629
Huang2012_EMTdown	265	23165231
Huang2012_EMTup	229	23165231
Huang2012_MED12kd_MEKiResist_54genes	54	23165231
VanDerFlier2007_WNTsig_DwAftDomNegTCFexpr	15	17320548
DeSousa2013_CCS3_Serrated_46genes	46	23584092
colon.MEKiSS.s32_down	25	
colon.MEKiSSsecreted.s32_down	25	
KRAS.dependency.signature_up	33	
Dry.signature_up	17	
Dry.signature_down	13	
EMT_LiteratureMarkers_up	5	
EMT.Taube.Weinberg.GSE24202_up	93	20713713
EMT.Taube.Weinberg.GSE24202_down	156	20713713
HippoPWup.YAPtransfection.GSE10196_down	43	18413746
BreastCa.PoorPrognosisConsensus.Teschendorff2006_up	15	17076897
p53-mut.Miller2005_up	5	16141321
PTENlossBreastCa.Saal2007_up	112	18066063
p53mut.BreastCa.Troester2006_up	32	17150101
Epithelial.markers.Literature_up	34	
Verhaak2010.Proneural_up	137	20129251
Verhaak2010.Proneural_down	73	20129251
Verhaak2010.Mesenchymal_down	46	20129251
Prat_ClaudinLow_up	425	20813035
Prat_ClaudinLow_down	357	20813035
S7_mDCs_plus_Monocytes	15	
S10_B_cells	50	
S12_T_cells_aCD3_aCD28_activated	50	
Bennett2003.SLE.granulopoiesis.signature	20	12642603
Bennett2003.SLE.ifn.sig	26	12642603
Chaussabel2008_M1.2_ifn.sig	27	18631455
Chaussabel2008_M3.4_ifn.sig	53	18631455
Chaussabel2008_M5.12_ifn.sig	59	18631455
Rice2013_IFNsig_6g	6	24183309
Walsh2007_IFNsig_6g	6	17968926

PlasmaCell_signature_24g	24	
Budinska2013.deregulated.in.CRC	27	23836465
Budinska2013.chromosome20q.CRC	33	23836465
Budinska2013.proliferation	83	23836465
Budinska2013.colon.crypt.markers.CRC	16	23836465
Budinska2013.EMT.stroma.CRC	310	23836465
Budinska2013.immune.response.CRC	102	23836465
Wright2003_ABC_DiffuseLargeBcellCarcinoma	21	12900505
Speers2015_BrCa_radioresistance_down	28	25904749
Lehmann.2011.BL2.up.refined	23	21633166
Lehmann.2011.IM.up.refined	174	21633166
IFNsig.Staub2015	7	
Walter2013_HNCa_atypical_high	132	23451093
Walter2013_HNCa_classical_high	62	23451093
Walter2013_HNCa_mesenchymal_high	246	23451093
MasqueSoler2013_DLBCL_nonMolBL	4	24030260
MasqueSoler2013_DLBCL_molBL	6	24030260
MasqueSoler2013_DLBCL_ABC	11	24030260
Scott2014_DLBCL_ABC	8	24398326
YAP_target_genes	8	
DNArepairScore_high_Kang2012	19	22505474
DNArepairScore_low_Kang2012	4	22505474
DDR_genes	43	
Mulligan2014_DDRD_up	24	24402422
Mulligan2014_DDRD_Group3	7	24402422
Mulligan2014_DDRD_Group4	10	24402422
Hou2010_LuCa_SCC	47	20421987
Hou2010_LuCa_ADC	5	20421987
radio_sensitivity_genes_up_BMC_Kim2012	10	22846430
radio_sensitivity_genes_down_BMC_Kim2012	21	22846430
radioresistance_PNAS_Khodarev2004	51	14755057
YAP_20gSig_Staub2016	20	
YAP_23gSig_Staub2016	23	
YAP_48gSig_Staub2016	48	
DDR_Alt-NHEJ	4	
DDR_FA (Fanconi anemia pathway)	37	
DDR_HR (Homologous Recombination)	52	
DDR_MMR	26	
Platinum_sensitivity_JNCI2012	23	22505474
Topotecan_sig_Pitroda_2014	12	24670686
RPS_Pitroda_2014	4	28341751
PARPi_Deamon_2012	7	22875744

IFN_signature

ADAR, CCR2, CIC, CXCL10, FADS1, FCGR1A, IFI27, IFI44, IFI6, IFIT1, IFIT2, IFIT3, IFIT5, IL1RN, ISG15, LGALS3BP, LY6E, MARCKS, MX1, MX2, OAS1, OAS2, OAS3, OASL, PLSCR1, RASGEF1B, RNASE2, SERPING1, SOCKS1, STAT1, TNFSF10, XAF1

KinetochoreNet
BUB1, BUB1B, CALCOCO1, KNL1, CBX3, CBX5, CENPE, CENPH, CETN3, DSN1, E2F1, E2F4, FOXO1, HNF1A, HNF4A, KDM5B, KLHL12, MIS12, NDC80, NEK2, NSL1, NUF2, PMF1, PSMC2, RB1, SMC1A, SPC24, SPC25, UBR5, USHBP1, ZW10, ZWINT

B_cell_signature_IRIS
ALG5, AMPD1, AP1, B4GALT3, TNFRSF13C, BANK1, BCL11A, TNFRSF17, FAM129C, BLK, BLNK, BMP8B, STAP1, VCPKMT, MYDGF, EDEM2, C21orf83B, CD19, MS4A1, CD79A, CD79B, KLF6, CPNE5, CXCR5, DDOST, DKFZp667L0210, DTNB, EAF2, EIF2AK3, ELL2, ERN1, PDIA4, EST, FBH1, FCRL1, FCRL2, FKBP11, TENT5C, TXNDC15, TMEM156, EME1, DERL3, FCRLA, GNG7, GPRC5D, PLPP5, FCRL5, SPCS2, FAM30A, SEL1L3, KLHL14, LOC220213, LOC51061, LZTFL1, MAN1A1, MANEA, NXPE3, MT-ND6, NLRP7, NGLY1, OSBPL10, PACAP, PAX5, PC4, PNOC, POU2AF1, QRSL1, RALGPS2, RPN1, SCFD1, SEC24A, KDM5D, SPATS2, SPIB, SSR1, HSPA13, TCF3, TCL1A, SEC62, TLR10, HSP90B1, TRAM1, TRAM2, TXNDC5, UBE2G1, UBE2J1, Ufm1, EZR, VPREB3, WNT10A

T_cell_signature_IRIS
BCL11B, CD3D, CD3E, CD3G, CD5, CD6, CD8A, CD8B, CTLA4, CXCR6, IL17F, IL22, IL9, TRA

DNAsynthesisFuncNet
APEX1, CCNA2, CCND1, CCND2, CCND3, CCNE2, CCNG1, CDC25C, CDK2, CDK4, CDK5, CDK6, CDK7, CDKN1A, CDKN1B, CDKN1C, CDT1, CHAF1A, CHTF18, DNMT1, DUSP1, EP300, FEN1, FZR1, HDAC1, HELB, HNRNPA1, PCLAF, LIG1, MRE11, NBN, NEIL1, NEIL2, PARP1, PCNA, POLA1, POLB, POLD1, POLD2, POLD3, POLD4, POLDIP2, POLE, POLH, POLI, POLM, RAD18, RAD9A, RBL1, RBL2, RECQL4, REV1, RFC2, RFC3, RPA1, RUVBL2, SKP2, TERT, TYMS, WRN, XRCC1, XRCC5, XRCC6, YBX1

CDK8_genomic_neighbors
NUP58, RNF6, CDK8, GPR12, USP12, RASL11

EMTup.QiagenPCRarray
AHNAK, BMP1, CALD1, CAMK2N1, CDH2, COL1A2, COL3A1, COL5A2, FN1, FOXC2, GNG11, GSC, IGFBP4, ITGA5, ITGAV, MMP2, MMP3, MMP9, MSN, SERPINE1, SNAI1, SNAI2, SNAI3, SOX10, SPARC, STEAP1, TCF4, TIMP1, TMEFF1, TMEM132A, TWIST1, VCAN, VIM, VPS13A, WNT5A, WNT5B

Cytosolic.Ribosomal.Proteins
RPL10, RPL10A, RPL11, RPL12, RPL13, RPL13A, RPL13P5, RPL14, RPL15, RPL17, RPL18, RPL18A, RPL21, RPL22, RPL23, RPL24, RPL26, RPL27A, RPL28, RPL29, RPL3, RPL30, RPL31, RPL32, RPL35, RPL35A, RPL36, RPL37, RPL37A, RPL39L, RPL3L, RPL4, RPL5, RPL6, RPL7, RPL7A, RPL8, RPLP1, RPLP2, RPS10, RPS11, RPS12, RPS13, RPS14, RPS15, RPS15A, RPS16, RPS17, RPS18, RPS19, RPS2, RPS20, RPS21, RPS25, RPS26, RPS27, RPS27A, RPS27L, RPS28, RPS29, RPS3, RPS3A, RPS4Y1, RPS5, RPS6, RPS7, RPS8, RPS9

SomaLogic_PlateletActivationInPlasma
BDNF, TIMP3, CCL5, MMP9, PF4, ANGPT1, MDK, SERPINE1, SPARC, APP, CTSA, SERPINE2, DKK4, THBS1, PDGFB

ECM_qPCR_panel_Qiagen
ADAMTS13, ADAMTS8, MMP1, MMP10, MMP11, MMP12, MMP13, MMP14, MMP15, MMP16, MMP2, MMP3, MMP7, MMP8, MMP9, SPG7, TIMP1, CD44, CDH1, CLEC3B, CNTN1, COL11A1, COL12A1, COL14A1, COL15A1, COL16A1, COL1A1, COL4A2, COL5A1, COL6A1, COL6A2, COL7A1, COL8A1, FN1, ANOS1, THBS1, TIMP2, TIMP3, CTGF, CTNNA1, CTNNB1, CTNND1, CTNND2, VCAN, ECM1, HAS1, SPP1, TGFBI, THBS2, THBS3, TNC, VTN, ICAM1, ITGA1, ITGA2, ITGA3, ITGA4, ITGA5, ITGA6, ITGA7, ITGA8, ITGAL, ITGAM, ITGAV, ITGB1, ITGB2, ITGB3, ITGB4, ITGB5, LAMA1, LAMA2, LAMA3, LAMB1, LAMB3, LAMC1, NCAM1, PECAM1, SELE, SELL, SELP, SGCE, SPARC, VCAM1

colon.MEKiSS.s32_down
C3, MMP9, DCN, SERPINF1, MGP, C1S, CDH11, SNORD114-3, ISLR, CTSK, MYH11, IL3RA, SULF1, ANTXR1, LUM, FBLN5, THBS2, C1R, ACTA2, IGFBP5, MXRA5, APOD, GUCY1A1, BGN, CRISPLD2

colon.MEKiSSsecreted.s32_down
C3, MMP9, SERPINF1, DCN, IGFBP5, ISLR, MMP2, LUM, MGP, FBLN5, AEBP1, A2M, MFAP4, ASPN, SPARCL1, OLFML2B, CRISPLD2, TIMP3, BGN, SRPX2, COL6A1, APOD, CXCL12, SPARC, AOAH

KRAS.dependency.signature_up
SYK, ESRP1, ST14, TMEM30B, SPINT1, RAB25, KDF1, GRHL2, GALNT3, SCNN1A, MPZL2, ITGB6, IRF6, INPP4B, PCDH1, C6orf141, HS3ST1, CDS1, DNAJA4, F11R, PROM2, CLDN7, C1orf116, SCEL, SCIN, S100A14, ANKRD22, MAL2, EHF, MSRB3, INAVA, TTC9, DENND1C

Dry.signature_up
ZNF106, PROS1, LZTS1, TRIB2, DUSP4, ETV4, ETV5, DUSP6, PHLDA1, SPRY2, ELF1, LGALS3, FXYD5, S100A6, SERPINB1, SLCO4A1, MAP2K3

Dry.signature_down
IL6, CD274, G0S2, STAC, COL5A1, COL12A1, SERPINE1, CRIM1, LOX, GPR176, FZD2, BASP1, CLU

EMT_LiteratureMarkers_up
SNAI1, SNAI2, TWIST1, VIM, CDH2

Epithelial.markers.Literature_up
SH2D3A, JUP, RAB25, CDH1, LSR, SPINT2, DDR1, GRHL2, GALNT3, CDS1, MAL2, CRB3, EPCAM, LLGL2, LAD1, TMEM125, PRSS8, SFN, ELF3, C1orf116, OCLN, PPL, INAVA, MAP7, ARHGEF5, S100A14, CDH3, MACC1, CHMP4C, HOOK1, CBLC, DSC2, PLS1, MAP3K9

S7_mDCs_plus_Monocytes
GCH1, CMPK2, ISG15, CXCL10, CXCL9, IFI6, EPSTI1, IL15RA, BATF3, IL15, APOL3, IFI44, IFIH1, ZNRF1, LOC100506459

S10_B_cells
BLK, CD79A, CD79B, CXCR5, FCRL1, P2RX5, FAM30A, POU2AF1, VPREB3, PCDH9, FCRL5, QSOX2, FCRLA, KLHL14, SGCE, IGHM, DSP, CCDC191, PAX5, PEG10, IGLJ3, STAG3, BTLA, LOC100130458, EML6, SLC38A11, CD19, CPNE5, CD24, SNX22, CD22, STRBP, CD200, PIK3C2B, STAP1, SYBU, CNTNAP2, IGLL3P, LARGE2, LINC00926, HIP1R, DTX1, LOC100507616, PLEKHG1, MACROD2, ABCB4, GGA2, IGHD, HLA-DOB, PLPP5

S12_T_cells_aCD3_aCD28_activated
CCNA2, PBK, PTTG1, SAAL1, ZWINT, UBE2T, MAD2L1, CXCR6, UTP15, DEPDC1B, MELK, CDCA7, NCAPG2, CENPH, FIGNL1, NDC80, CDKN3, ZC3HAV1L, POLE2, LAG3, SLC25A17, CENPM, COL6A3, HPGD, TIPIN, RMI2, NUF2, NCAPH, RTTN, RRM2, MCM2, PCLAF, TMEM200A, WDR89, TMEM135, NPM3, ZBTB9, FAM83D, TYMS, CENPK, GPR171, CHAF1B, NECTIN3, GRPEL2, KLC2, DCLRE1A, SLC9B2, C5orf30, WEE1, ABCD2

PlasmaCell_signature_24g
PDIA6, PRDM1, MAN1A2, RABAC1, CAV1, IGF1, HYOU1, HSPA13, CD38, ELL2, UAP1, SDC1, B9D1, STT3A, IGLV1.44, MYDGF, WFS1, PDIA4, RRBP1, GFPT1, TNFRSF17, MAN1A1, HERPUD1, RWDD2A

IFNsig.Staub2015
IFIT3, IFIT2, IFIT1, IFI44, IFI44L, OASL, OAS3

YAP_target_genes
CTGF, CYR61, ANKRD1, FOSL1, ACTN1, PDLIM7, AXL, ODC1

DDR_genes
AKT1, ATM, ATR, BAP1, BARD1, BRCA1, BRCA2, BRIP1, CDK12, CHEK1, CHEK2, CTNNB1, ERCC4, ABRAXAS1, FANCA, FANCD2, FANCE, FANCI, FANCL, KRAS, MLH1, MRE11, MSH2, MSH6, MUTYH, NBN, PALB2, PIK3CA, PPP2R2A, PTEN, RAD50, RAD51, RAD51B, RAD51C, RAD51D, RAD52, RAD54B, RAD54L, RPA1, TP53, TP53BP1, XRCC2, XRCC3

YAP_20gSig_Staub2016

AMOTL2, GPRC5A, TLCD2, TJP1, BCL9L, AJUBA, SDC4, RBMS2, CRIM1, TNFRSF12A, EPHA2, FHL2, FOSL1, ANXA2, CYR61, MYOF, CAVIN1, RND3, LOC100288911, NTN4

YAP_23gSig_Staub2016
AJUBA, AMOTL2, ANXA2, AXL, BCL9L, BOK, CRIM1, CYR61, DCBLD2, EPHA2, FHL2, FOSL1, GPRC5A, MYOF, NTN4, CAVIN1, RBMS2, RND3, SDC4, TJP1, TLCD2, TNFRSF12A, YAP1

YAP_48gSig_Staub2016
AMOTL2, GPRC5A, TLCD2, TJP1, BCL9L, AJUBA, SDC4, RBMS2, CRIM1, TNFRSF12A, EPHA2, FHL2, FOSL1, ANXA2, CYR61, MYOF, CAVIN1, RND3, LOC100288911, NTN4, ITGA3, DCBLD2, AXL, CAV1, AHNAK2, TGFBI, MT2A, CAV2, TNFAIP1, RTN4, TIMP2, YAP1, ERBB2, TUFT1, EDN1, CLIC3, ATP8B1, SSH3, C6orf132, DSP, KRT19, SERINC2, KIAA1522, BOK, RHOD, PPP1R13L, F3, RHPN2

DDR_Alt-NHEJ
LIG1, LIG3, PARP1, XRCC1

DDR_FA (Fanconi anemia pathway)
CENPS, BARD1, BLM, BRCA1, BRCA2, BABAM2, BRIP1, ABRAXAS1, DNA2, FAAP100, FAAP24, FAN1, FANCA, FANCB, FANCC, FANCD2, FANCE, FANCF, FANCG, FANCI, FANCL, FANCM, HELQ, HES1, KAT5, PALB2, RAD51, RAD51C, RMI2, STRA13, BHLHE40, TELO2, TOP3A, TOP3B, UBE2T, USP1, WDR48

DDR_HR (Homologous Recombination)
BLM, BRCA1, BRCA2, EID3, EME1, EME2, GEN1, H2AFX, HELQ, HFM1, KAT5, MRE11, MUS81, NBN, NSMCE3, NFATC2IP, NSMCE1, NSMCE2, NSMCE4A, PARG, PAXIP1, PPP4C, PPP4R1, PPP4R2, PPP4R4, RAD50, RAD51, RAD51B, RAD51C, RAD51D, RAD52, RAD54B, RAD54L, RDM1, RECQL, RECQL4, RECQL5, RMI2, RPA1, RPA2, RPA3, RPA4, SEM1, SLX1A, SLX4, SMC5, SMC6, SPO11, TOP3A, TOP3B, UIMC1, WRN

DDR_MMR
EXO1, HMGB1, LIG1, MLH1, MLH3, MSH2, MSH3, MSH4, MSH5, MSH6, PCNA, PMS1, PMS2, POLD1, POLD2, POLD3, POLD4, RFC1, RFC2, RFC3, RFC4, RFC5, RPA1, RPA2, RPA3, RPA4
